# Th17 micro-milieu regulates NLRP1-dependent caspase-5 activity in skin autoinflammation

**DOI:** 10.1371/journal.pone.0175153

**Published:** 2017-04-19

**Authors:** Stephanie Zwicker, Eva Hattinger, Daniela Bureik, Aleksandra Batycka-Baran, Andreas Schmidt, Peter-Arne Gerber, Simon Rothenfusser, Michel Gilliet, Thomas Ruzicka, Ronald Wolf

**Affiliations:** 1 Department of Dermatology and Allergology, Ludwig-Maximilian University Munich, Frauenlobstr. 9–11, Munich, Germany; 2 Department of Dental Medicine, Karolinska Institute, Alfred Nobels Allé 8, Huddinge, Sweden; 3 Department of Dermatology, Venereology and Allergy, Wroclaw Medical University, Chalubinskiego 1, Wroclaw, Poland; 4 Division of Clinical Pharmacology, Medizinische Klinik IV, Ludwig-Maximilian University Munich, Ziemssenstr. 1, Munich, Germany; 5 Department of Dermatology, University Hospital Düsseldorf, Moorenstrasse 5, Düsseldorf, Germany; 6 Department of Dermatology, University Hospital of Lausanne, CHUV University Hospital, Rue du Bugnon 46, Lausanne, Switzerland; 7 Department of Dermatology and Allergology, Philipps University Marburg, Marburg, Germany; Universite Paris-Sud, FRANCE

## Abstract

IL-1β is a potent player in cutaneous inflammation and central for the development of a Th17 micro-milieu in autoinflammatory diseases including psoriasis. Its production is controlled at the transcriptional level and by subsequent posttranslational processing via inflammatory caspases. In this study, we detected inflammatory caspase-5 active in epidermal keratinocytes and in psoriatic skin lesions. Further, interferon-γ and interleukin-17A synergistically induced caspase-5 expression in cultured keratinocytes, which was dependent on the antimicrobial peptide psoriasin (S100A7). However, diseases-relevant triggers for caspase-5 activity and IL-1β production remain unknown. Recently, extranuclear DNA has been identified as danger-signals abundant in the psoriatic epidermis. Here, we could demonstrate that cytosolic double-stranded (ds) DNA transfected into keratinocytes triggered the activation of caspase-5 and the release of IL-1β. Further, interleukin-17A promoted caspase-5 function via facilitation of the NLRP1-inflammasome. Anti-inflammatory vitamin D interfered with the IL-1β release and suppressed caspase-5 in keratinocytes and in psoriatic skin lesions. Our data link the disease-intrinsic danger signals psoriasin (S100A7) and dsDNA for NLPR1-dependent caspase-5 activity in psoriasis providing potential therapeutic targets in Th17-mediated skin autoinflammation.

## Introduction

Activation of the Th17 pathway has been linked to several autoimmune diseases including the skin. Psoriasis is a autoinflammatory skin disease of unknown origin that affects two percent of the population [1]. In psoriasis, interleukin-1β (IL-1β) is an important player for the development of the inflammatory Th17 phenotype [2]. Systemic interference with IL-1β shows beneficial effects in clinical trials with patients suffering from psoriasis, specifically in the inflammatory manifestations of psoriasis [3]. Accordingly, topical interference with IL-1β, such as by anti-psoriatic vitamin D analogues, is of therapeutic interest [4].

In psoriatic lesions, keratinocytes are a major source of active IL-1β [1, 5]. Pro-inflammatory mediators like TNFα up-regulate IL-1β through NF-κB signaling on transcriptional level [6]. To be functional, IL-1β precursors need to be activated by proteolytic cleavage and a constitutively enhanced IL-1β maturation alone can cause skin inflammation [7]. IL-1β maturation is mediated by inflammasomes, which can activate inflammatory caspases upon recognition of certain molecular patterns [8]. In psoriasis, caspase-1 is active in epidermal keratinocytes and has been linked to IL-1β production via ASC-dependent inflammasome complexes, such as NLRP3 and AIM2 [5, 9]. In comparison, NLRP1 is capable to additionally utilizes inflammatory caspase-5 for IL-1β activation independently of ASC[10–12]. So far, pathogen-associated patterns relevant for the defense against invading microbes in infectious diseases are known to activate the NLRP1 inflammasome, such as muramyl dipeptide (MDP) and lipopolysaccharide (LPS) [13, 14]. Recent studies reveal that NLRP1 gene variants confer susceptibility to non-infectious skin-associated autoinflammatory and autoimmune diseases, including vitiligo, lupus erythematosus, and psoriasis [15–18]. Accordingly, the enhanced expression of NLRP1and NLRP1-specific caspase-5 have been linked to the pathogenesis of Th17-driven skin diseases [12, 19–21]. Therefore, the identification of associated cytokine and intrinsic danger-associated molecular patterns that regulate caspase-5 activity in sterile chronic inflammatory diseases could lead to novel therapeutic approaches that target these patterns.

Intracellular self-DNA is physiologically encapsulated into the nucleus and mitochondria. Under inflammatory conditions, self-DNA can be detected in the cytosol of keratinocytes in sterile autoinflammatory diseases, including psoriasis [9, 22]. When cytosolic, self-DNA becomes a trigger, such as to activate the AIM2 inflammasome complex [23].

S100 peptides are another group of danger signals that have been originally discovered as antimicrobial peptides (AMP) [24, 25]. Psoriasin (S100A7) is increased in the psoriatic epidermis and exerts diverse immune-stimulating functions in Th17-mediated chronic inflammatory diseases, such as induction of proinflammatory cytokines and leukocyte chemotaxis [26, 27]. Here, we investigate regulators and triggers for epidermal NLRP1-dependent caspase-5 activation in a psoriasis-relevant cytokine micro-milieu. This study identifies novel molecular targets for approaches in Th17-mediated diseases using psoriasis as an example.

## Materials and methods

### Patients and skin samples

The study was conducted according to the Declaration of Helsinki Principles. Sample acquisitions were approved by the local ethical committee, Faculty of Medicine, Ludwig-Maximilian University, Munich, Germany. For all the procedures, informed patient’s written consent was obtained. Patients suffering from plaque psoriasis did not receive systemic therapy and no topical treatment for at least four weeks before entering the study. 4-mm punch biopsies were taken from a marker psoriatic plaques before treatment with a topical calcipotriol preparation (LEO Pharma, Neu-Isenburg, Germany) containing ointment (0.005%; applied twice daily) and 5 to 7 days after treatment onset. Skin biopsies from untreated lesional skin from psoriasis patients were collected and compared with biopsies from healthy volunteers. The biopsies were directly transferred to 1 ml TRIzol® (Invitrogen, Karlsruhe, Germany) for RNA extraction or snap frozen in liquid nitrogen for immunofluorescence staining.

### Human keratinocyte cell culture and stimulation

Normal human epidermal keratinocytes were cultured in EpiLife^®^ cell culture medium (Invitrogen, Carlsbad, CA, USA) supplemented with 0.06 mM calcium with 10 μg/ml gentamicin, 0.25 μg/ml amphotericin B, and growth serum (Invitrogen, Carlsbad, CA, USA) in a humidified atmosphere of 5% CO_2_ at 37°C. Cells were stimulated with IFNγ (100 ng/ml; Biomol, Hamburg; Germany), TNFα (50 ng/ml; Biomol, Hamburg; Germany), IL-17A (10 ng/ml; R&D Systems, Minneapolis, MN; USA), 1,25-dihydroxyvitamin D_3_ (1,25D_3_; 10^-9^M; 10^-8^M; Sigma, Steinheim, Germany), in some settings six hours prior to transfection with undigested or DNase-treated dsDNA [Poly(dA:dT)] (1 μg/ml; Sigma–Aldrich, St. Louis, MO, USA) for 18 hours. Normal human keratinocytes were transfected with siRNA oligonucleotides (5nM) targeting caspase-1, caspase-5, NLRP1, psoriasin (S100A7) or an non-target control using RNAiMAX transfection reagent according to manufacturer’s protocol (Invitrogen, Carlsbad, CA, USA): control, 5’-cgc gua agg ucg aau gca uaa tt-3’; caspase-1, 5’-gaa gac uca uug aac aua utt -3’; caspase-5, 5’-cca ccu aau gga aau auu utt-3’; NLRP1, 5’-gga gaa ucg agg aca uuu att-3’; psoriasin, 5’-gac aug uuu cac aaa uac att-3’. Subsequently after 48 hours, cells were stimulated as described above and either harvested for RNA or protein analysis.

### RNA isolation and quantitative real-time -PCR

Total RNA was isolated (Quick-RNA MiniPrep^TM^; HISS Diagnostics, Freiburg, Germany) and reversely transcribed (DyNAmo DNA Synthesis Finnzymes, Espoo, Finland) from epidermal keratinocytes according to the manufacturer’s instructions. Expression of IL-1β, caspase-1, caspase-5, NLRP1, NLRP3 and AIM2 was analyzed by SYBR Green supermix in CFX96-real-time detection system (Bio-Rad Laboratories, Hercules, CA, USA), calculated with the ΔC_T_ method [28] and compared to housekeeping genes, *β*-actin or PBGD (Qiagen, Hilden, Germany). Results are shown as fold induction of healthy tissue or unstimulated conditions.

### Enzyme-linked immunosorbent assay (ELISA)

Primary human keratinocytes were stimulated with IFNγ (100 ng/ml), TNFα (50 ng/ml), IL-17A (10 ng/ml), S100A7 (100 ng/ml) and transfected with [Poly(dA:dT)] (1 μg/ml; Sigma–Aldrich, St. Louis, MO, USA). Cell culture supernatants were collected after 24 hours and IL-1β levels were determined by IL-1β ELISA assay according to the manufacturer’s protocol (IL-1β ELISA Duo Set; R&D Systems, Minneapolis, MN, USA). Released IL-1β levels were normalized to total protein concentration in the culture supernatant using bovine serum albumin as a standard (562 nm, Nanophotometer, Implen, Munich, Germany) and shown as relative units combining repeated experiments.

### Immunoblot analysis

Total protein of skin samples, cell culture lysates and precipitated supernatants was prepared using T-PER Tissue Protein extraction lysis buffer (Thermo Fisher Scientific, Bonn, Germany) and quantified by BCA Protein Assay kit (Thermo Fisher Scientific, Bonn, Germany). Equivalent amounts of proteins (10–15 μg) were separated using a 12% SDS-polyacrylamid gel (Invitrogen, Carlsbad, CA, USA), transferred to reinforced nitrocellulose membranes and blocked (Tris buffered saline, pH 7.5, 0.1% Tween 20 (TBS-T), 5% milk powder) for 1 hour at room temperature. Membranes were incubated with anti-caspase-1 IgG (1:1000), anti-caspase-5 IgG (all Cell Signaling Technology, Inc., Beverly, MA, USA; 1:1000), and anti-NLRP1(Nalpy1-4) IgG (Enzo Life Sciences Inc., Farmingdale, NY, USA; 1:1000) in 5% BSA/TBS-T at 4°C overnight. Gel loading was controlled by detecting β- actin signal with monoclonal antibodies (Cell Signaling, Technology, Inc., Beverly, MA, USA; 1:2000). After washing with TBS-T, blots were developed by incubation with horseradish-peroxidase-conjugated secondary antibodies (Cell Signaling Technology, Inc., Beverly, MA, USA; 1:10.000) for 1 h at room temperature and visualized with chemiluminescence method following the manufacturer’s protocol (Thermo Fisher Scientific, Bonn, Germany).

### Immunofluorescence staining

Immunofluorescence staining was performed on 8 μm serial frozen sections of healthy and psoriatic skin fixed for 5 minutes in acetone at -20°C. The sections were blocked in 10% normal goat serum, and incubated overnight with anti-caspase-5 (Cell Signaling Technology, Inc., Beverly, MA, USA; 1:50). The sections were then incubated with goat anti-rabbit IgG conjugated with Alexa Fluor^®^ 647 (Invitrogen, Carlsbad, CA, USA; 1:250), diluted in 10% normal goat serum and incubated for 1 hour at room temperature in a dark humidified chamber. Staining with secondary antibodies only was performed as a negative control. Sections were overlaid with ProLong Gold antifade reagent containing DAPI (Invitrogen, Carlsbad, CA, USA). Fluorescent stained tissues were imaged using a fluorescent microscope Zeiss ImagerZ1 (Zeiss, Jena, Germany) using a 12-bit CCD digital camera PCO PixelFly (PCO, Kelheim, Germany).

### Statistical analysis

Data were expressed as pooled means + SEM of three independent experiments conducted in triplicates for each condition. Comparative data were analysed with GraphPad Prism 5.01 software (La Jolla, CA, USA) using Student’s *t*-test and ANOVA. A *P*-value of less than 0.05 was considered statistically significant.

## Results

### Inflammatory caspase-5 is induced and activated in psoriatic skin lesions

**Fig 1:**
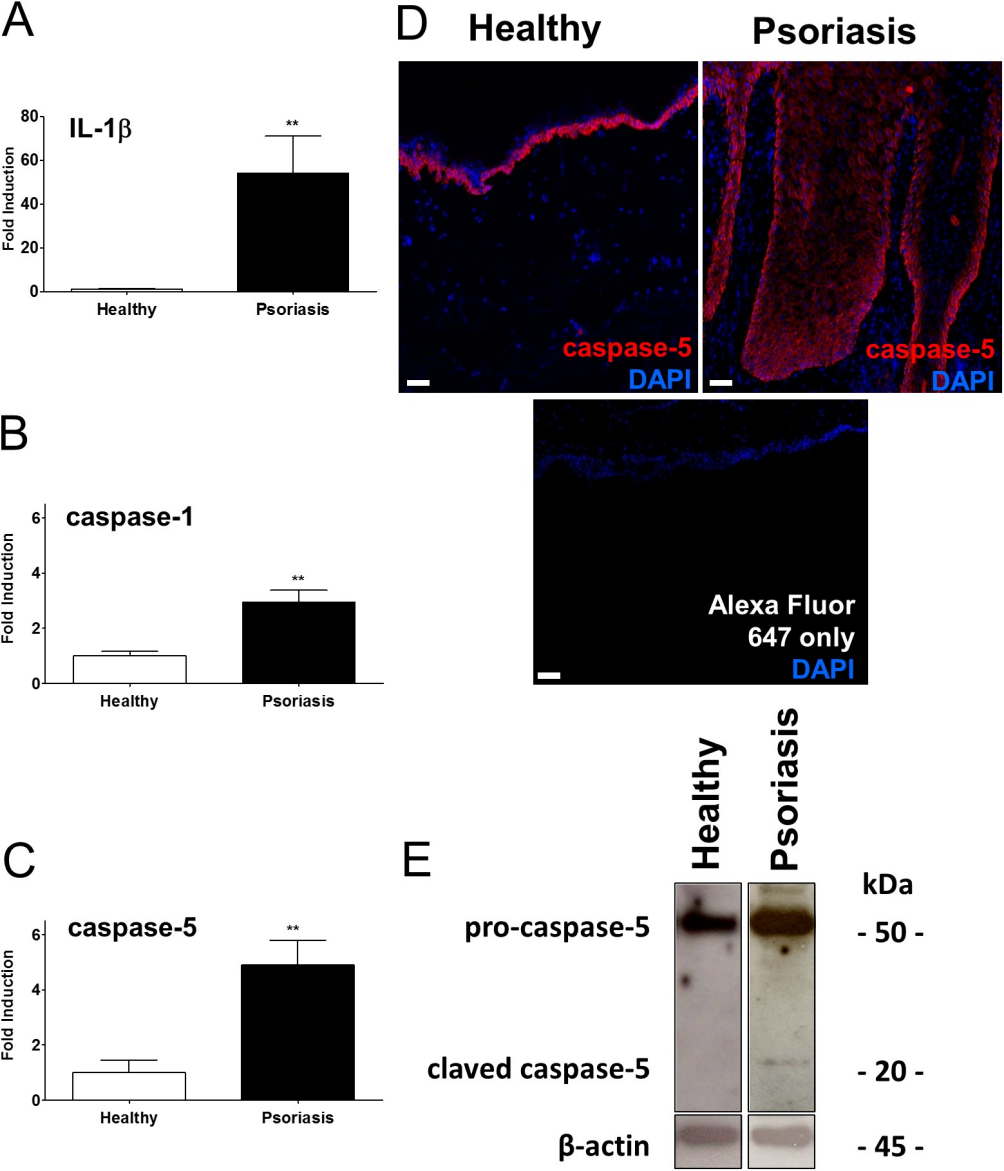
Inflammatory caspase-5 is increased and active in psoriatic skin. A-C, Increased expression of IL-1β, caspase-1, caspase-5 in healthy (H) and psoriatic (P) skin analyzed by RTqPCR and normalized to PBGD. Data represent mean + SEM **, *p* < 0.01, Student’s *t* test (n = 6–8). D, Immunofluorescent staining of caspase-5 in healthy skin which was enhanced in psoriasis (n = 3), scale bar = 50μm. Representative control section stained with secondary antibody (ab) only and DAPI. E, Representative immunoblot analysis of healthy and psoriatic skin stained for caspase-5, indicated are pro-caspase-5 and active caspase-5 in psoriasis compared to healthy skin versus β-actin (n = 3).

IL-1β has been identified as an important pro-inflammatory mediator increased in psoriatic skin lesions. To be functional, IL-1β precursors are cleaved into the biologically active form via inflammatory caspases [8]. Data revealed that transcripts of IL-1β, inflammatory caspase-1 and caspase-5 were up-regulated in psoriatic skin lesions (Fig 1A–1C). An epidermal expression and activation of IL-1β and caspase-1 has already been reported in psoriatic skin vs. normal but not for caspase-5[5, 9]. Here, immunofluorescent staining illustrated the increased expression of caspase-5 in psoriasis and showed a prominent distribution throughout the layers of the psoriatic epidermis compared to healthy skin (Fig 1D). Further, inflammatory pro-caspase-5 and cleaved caspase-5 could be detected in psoriatic lesions suggesting that caspase-5 activity is regulated by an autoinflammatory micro-milieu in epidermal keratinocytes in psoriasis (Fig 1E).

### Cytosolic dsDNA induces IL-1β release via caspase-5 by IFNγ-primed epidermal keratinocytes

**Fig 2:**
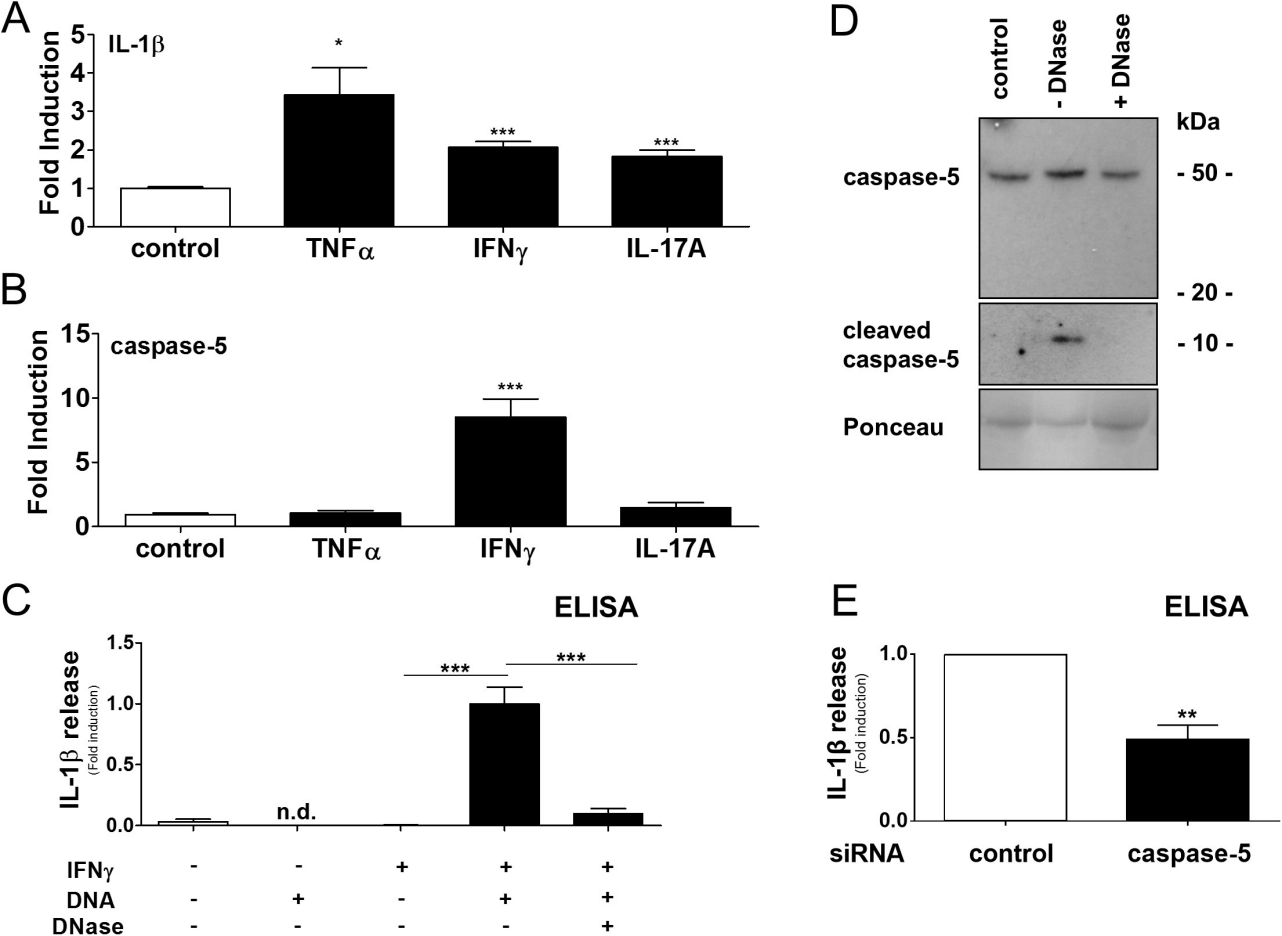
IFNγ and cytosolic dsDNA regulate inflammatory caspase-5 and IL-1β release by epidermal keratinocytes. A-B, Regulation of IL-1β and caspase-5 in Th1/Th17 cytokine-stimulated keratinocytes analyzed by RTqPCR and normalized to β-actin. Data represent mean + SEM, *, *p* < 0.05; **, *p* < 0.01; ***, *p* < 0.001, Student’s *t* test, n = 9. C, Keratinocytes stimulated with IFNγ, transfected with dsDNA [Poly(dA:dT)] ± DNase and the DNA-dependent IL-1β release was analyzed by ELISA. D, Representative immunoblotting of corresponding supernatants analyzed for DNA-dependent activation of caspase-5 (exposure times; caspase-5, 30s; cleaved caspase-5, 30min) compared to loading control (Ponceau staining), three independent experiments. E, Keratinocytes stimulated with IFNγ, transfected with dsDNA and indicated siRNA and the caspase-5 dependent IL-1β release was analyzed by ELISA. C, E, Data represent mean + SEM, **, *p* < 0.01; ***, *p* < 0.001, Student’s *t* test, n = 3–6.

To identify immune-regulatory factors for IL-1β and caspase-5 in the epidermis, keratinocytes were exposed to a psoriasis-relevant cytokines. Data showed that TNFα (2.1-fold) and IL-17A (1.8-fold) induce IL-1β but had no effect on inflammatory caspase-5. In comparison, IFNγ strongly increased caspase-5 expression besides IL-1β in epidermal keratinocytes (Fig 2A and 2B), however keratinocytes stimulated with IFNγ alone did not release IL-1β into the culture supernatant (Fig 2C). In inflamed psoriatic plaques, free DNA is detectable in the cytosol of keratinocytes [9], and we hypothesized that double-stranded (ds) DNA mediates caspase-5 activation and IL-1β release by keratinocytes. Subsequently, cultured cells primed with IFNγ and transfected with dsDNA [Poly(dA:dT)] led to IL-1β activation compared to single treatment. In this setting, transfection of DNase-treated dsDNA abolished the IL-1β release by keratinocytes confirming dsDNA as a trigger. Beside the activating effect of DNA, we could further show that cytosolic dsDNA induces a pro-IL-1β expression in keratinocytes but not caspase-5 (S1A Fig and S1B Fig). In addition, IFNγ-primed keratinocytes secreted higher amounts of active caspase-5 subunits (10 KDa bands) in response to cytosolic DNA (-DNase, Fig 2D). When dsDNA was treated with DNase prior transfection, these bands vanished (+DNase) indicating a DNA-dependent activation. To test the functional relevance, the investigated caspases were targeted by siRNA interference (Fig 2E). Data revealed that knock-down of caspase-5 inhibited the DNA-dependent IL-1β release by IFNγ-primed keratinocytes (caspase-5 siRNA interference efficacy, S2A Fig). Data suggest regulatory and activating factors in the psoriatic micro-milieu important for epidermal IL-1β production via inflammatory caspases.

### The antimicrobial peptide psoriasin (S100A7) mediates caspase-5 and IL-1β release

**Fig 3:**
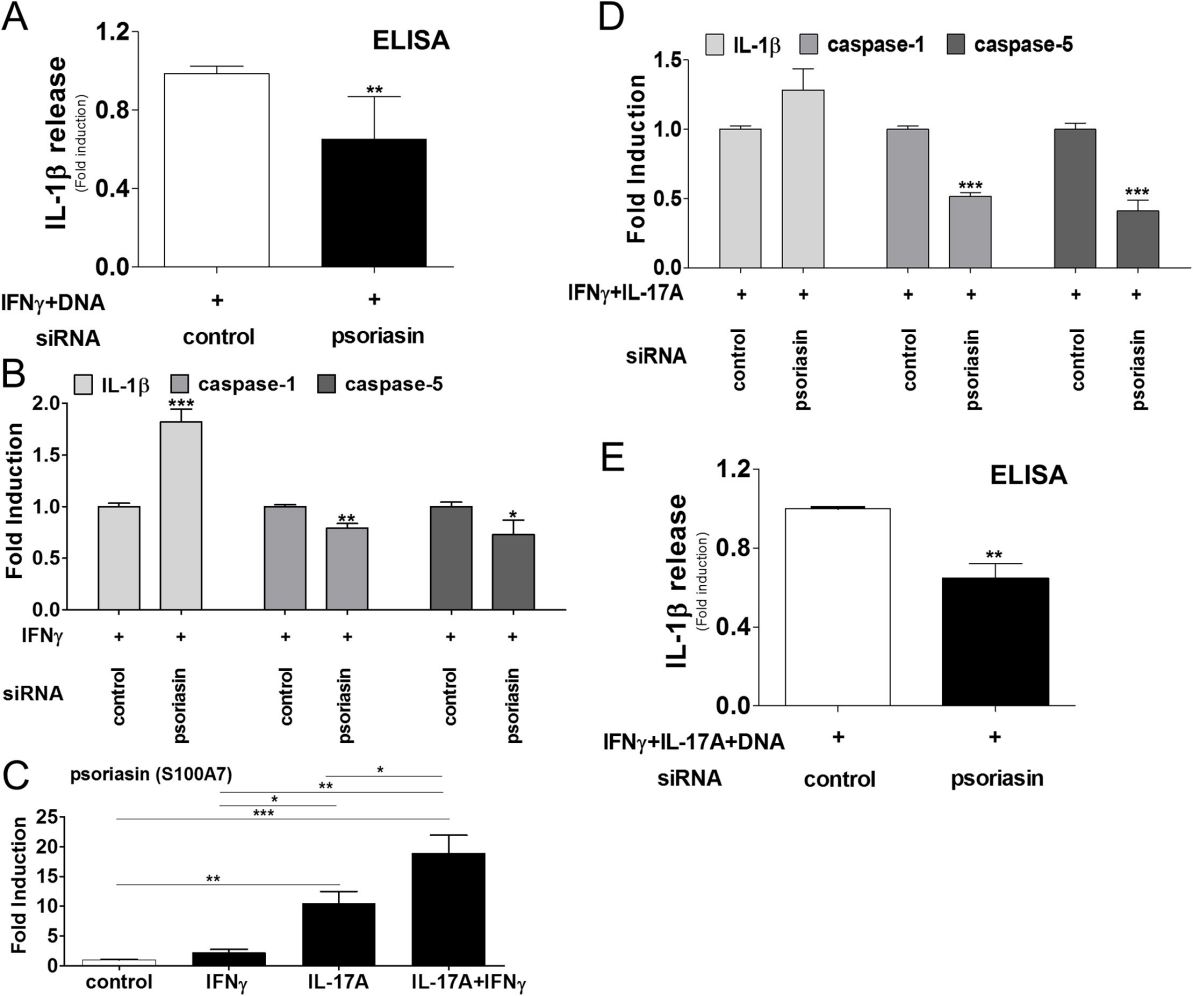
The antimicrobial peptide psoriasin (S100A7) mediates cytokine-dependent caspase regulation and IL-1β release by epidermal keratinocytes. A, E, Keratinocytes stimulated with IFNγ, IL-17A, transfected with dsDNA and indicated siRNA, and the psoriasin-dependent IL-1β release was analyzed by ELISA. Data represent mean + SEM, **, *p* < 0.01, Student’s *t* test, n = 7–8. B, D, Regulation of IL-1β, caspase-1, caspase-5 in cytokine-stimulated keratinocytes, transfected with psoriasin-targeting siRNA was analyzed by RTqPCR and normalized to β-actin. Data represent mean + SEM, *, *p* < 0.05; **, *p* < 0.01; ***, *p* < 0.001 determined by Student’s *t* test, n = 9. C, Induction of psoriasin in cytokine-stimulated keratinocytes analyzed by RTqPCR and normalized to β-actin. Data represent mean + SEM, *, *p* < 0.05; **, *p* < 0.01; ***, *p* < 0.001 determined by ANOVA, n = 9.

Psoriasin (S100A7) has been discovered as an antimicrobial peptide up-regulated in inflamed psoriatic skin [24, 29, 30]. IFNγ induces psoriasin release by keratinocytes [24], and we hypothesized that psoriasin is another intrinsic danger-associated molecular pattern that regulates IL-1β production. In IFNγ-primed keratinocytes, targeting psoriasin by siRNA interference suppressed the IL-1β release (0.7-fold) in response to cytosolic DNA (Fig 3A; psoriasin siRNA interference efficacy, S2B Fig). In this setting, psoriasin knock-down induced the expression ofpro-IL-1β but the antimicrobial peptide down-regulated IL-1β activating caspase-5 (0.7-fold) besides caspase-1 (0.8-fold) indicative for the overall suppressed IL-1β production in the presence of IFNγ (Fig 3B). However, the psoriatic epidermis is exposed to a mixed Th1/Th17 cytokine milieu, and IL-17A is a key cytokine in the disease pathogenesis. We could further show that IL-17A enhances the IFNγ-mediated psoriasin expression in keratinocytes suggesting a regulatory role here (Fig 3C). In keratinocytes treated with IFNγ and IL-17A, targeting psoriasin showed a slightly stronger regulatory effect on caspase-5 (0.4-fold) and caspase-1 (0.5-fold), whereas pro-IL-1β expression was not significantly altered (Fig 3D). Under mixed cytokine conditions, suppression of psoriasin inhibited the overall IL-1β production in response to cytosolic DNA (0.6-fold) (Fig 3E). Data suggest that psoriasin mediates the IFNγ and IL-17A induced regulation of inflammatory caspases and IL-1β production in human epidermal keratinocytes.

### IL-17A induces caspase-5 and facilitates NLRP1-mediated IL-1β release

**Fig 4:**
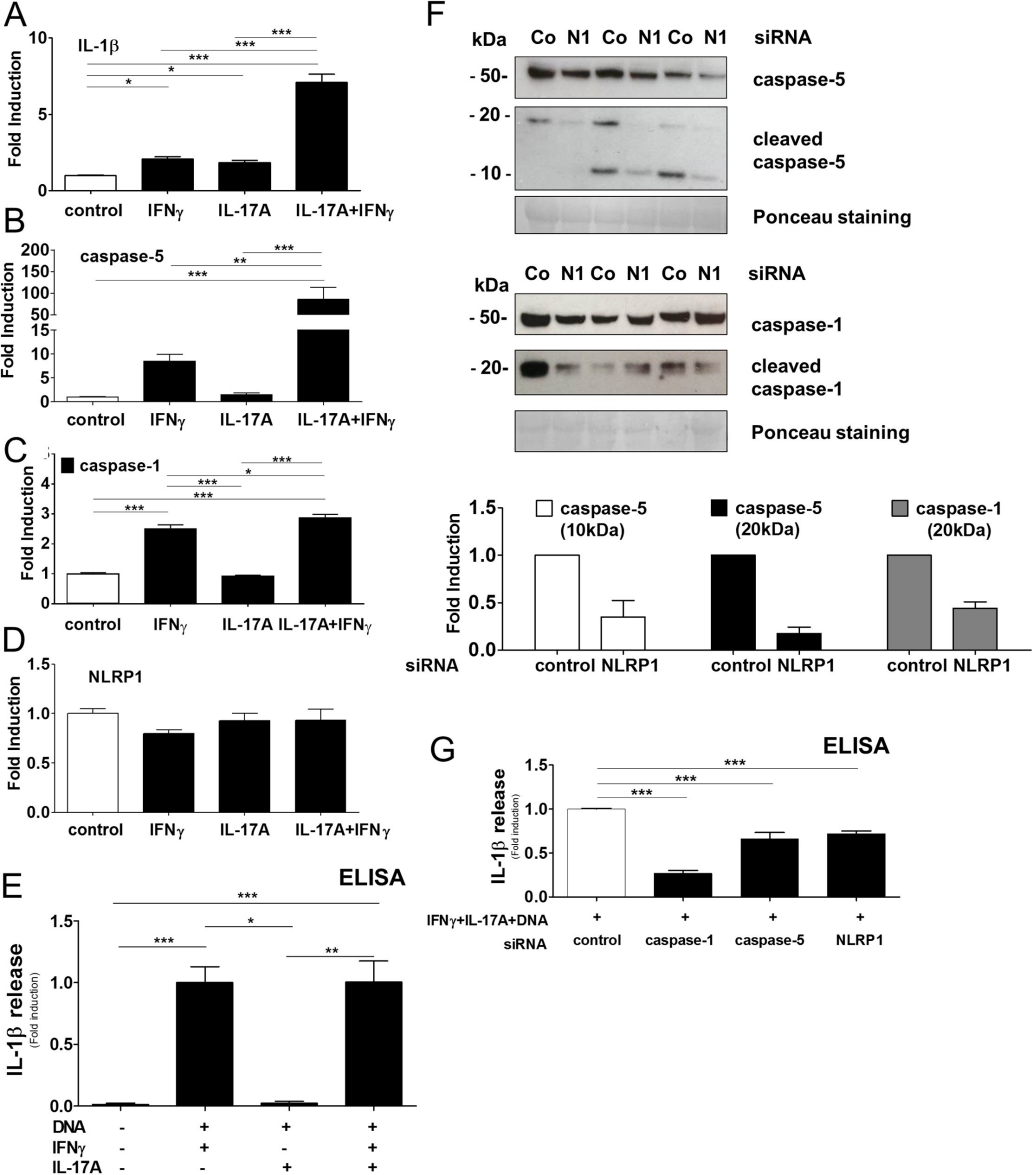
IL-17A amplifies caspase-5 induction and controls NLRP1-mediated IL-1β release by epidermal keratinocytes. A-D, Regulation of IL-1β, caspase-1, caspase-5, NLRP1in IFNγ- and IL-17A -stimulated keratinocytes analyzed by RTqPCR and normalized to β-actin. Data represent mean + SEM of three independent experiments performed in triplicates *, p < 0.05; **, *p* < 0.01; ***, *p* < 0.001 determined by ANOVA. E, dsDNA-transfected keratinocytes stimulated with IFNγ, IL-17A, and the cytokine-dependent IL-1β release was analyzed by ELISA. Data represent mean + SEM *, p < 0.05; *** p< 0.001 determined by ANOVA, n = 6. F, IFNγ and IL17A-treated keratinocytes, transfected with dsDNA and with siRNA targeting NLRP1 (N1) or non-coding siRNA (Co), and the supernatant was analyzed for NLRP1-dependent activation of caspase-5 (exposure time 20s; active p10, p20 subunits; exposure time, 120s) and caspase-1 (exposure time 35s). Corresponding protein levels were quantified by densitometry, n = 3 and compared to Ponceau staining (loading control). G, Keratinocytes stimulated with IFNγ, IL-17A transfected with dsDNA and indicated siRNA, and the NLRP1 inflammasome-dependent IL-1β release was analyzed by ELISA. Data represent mean + SEM, ***, p < 0.001 determined by ANOVA, n = 6.

Regarding the relevance of IL-17A in psoriasis, we investigated the caspase- mediated IL-1β activation by epidermal keratinocytes further. In IFNγ-primed keratinocytes, co-stimulation with IL-17A increased the expression of pro-IL-1β (Fig 4A). Under the combined stimulation, IL-17A amplified the IFNγ-mediated expression of caspase-5, whereas caspase-1 was only slightly induced (Fig 4B and 4C; S3A Fig and S3B Fig). Inflammatory caspases are functionally dependent on inflammasome complexes, and IL-17A interfered with the regulation of caspase-1 recipient NLRP3 and dsDNA-sensing AIM2, whereas the expression of NLRP1 which can additionally activate caspase-5, remained unaffected (Fig 4D; S3D Fig and S3E Fig). To analyze the mixed cytokine effect on IL-1β production, cultured keratinocytes were transfected with dsDNA and stimulated with IFNγ and IL-17A (Fig 4E). The combined stimulation induced an IL-1β release similar to treatment with IFNγ, whereas treatment of keratinocytes with IL-17A alone had no enhancing effect when compared to control. The regulatory data suggest that NLRP1-mediated caspase activity contributes to the IFNγ and IL-17A sustained IL-1β release, which was analyzed next. dsDNA-transfected and IFNγ + IL-17A primed keratinocytes secreted cleaved bands of activated caspase-5 (10kDa, 20kDa) and caspase-1 (20 kDa) into the culture supernatant (Co, control siRNA; Fig 4F). Under this condition, co-transfection of keratinocytes with siRNA targeting NLRP1 vanished these bands (N1, NLRP1siRNA; Fig 4F, NLRP1 siRNA interference efficacy; Figs 3C and S3) as quantified below. Further, knock-down of either NLRP1, associated caspase-5 or caspase-1 led to a significant decrease in IL-1β release by IFNγ and IL-17A-treated keratinocytes when transfected with dsDNA (0.7-fold, 0.7-fold, 0.3-fold, respectively) (Fig 4G; caspase-1 siRNA interference efficacy, S2C Fig). Data suggest that the mixed pro-inflammatory micro-milieu in Th1/Th17-mediated psoriasis provides a relevant setting for NLRP1-dependent inflammasome activation. Under these conditions, the dsDNA-sensing NLRP1 can contribute to IL-1β release besides other inflammasomes active in epidermal keratinocytes.

### Vitamin D interferes with IL-1β release by epidermal keratinocytes and suppresses caspase-5 in psoriatic skin lesions

**Fig 5:**
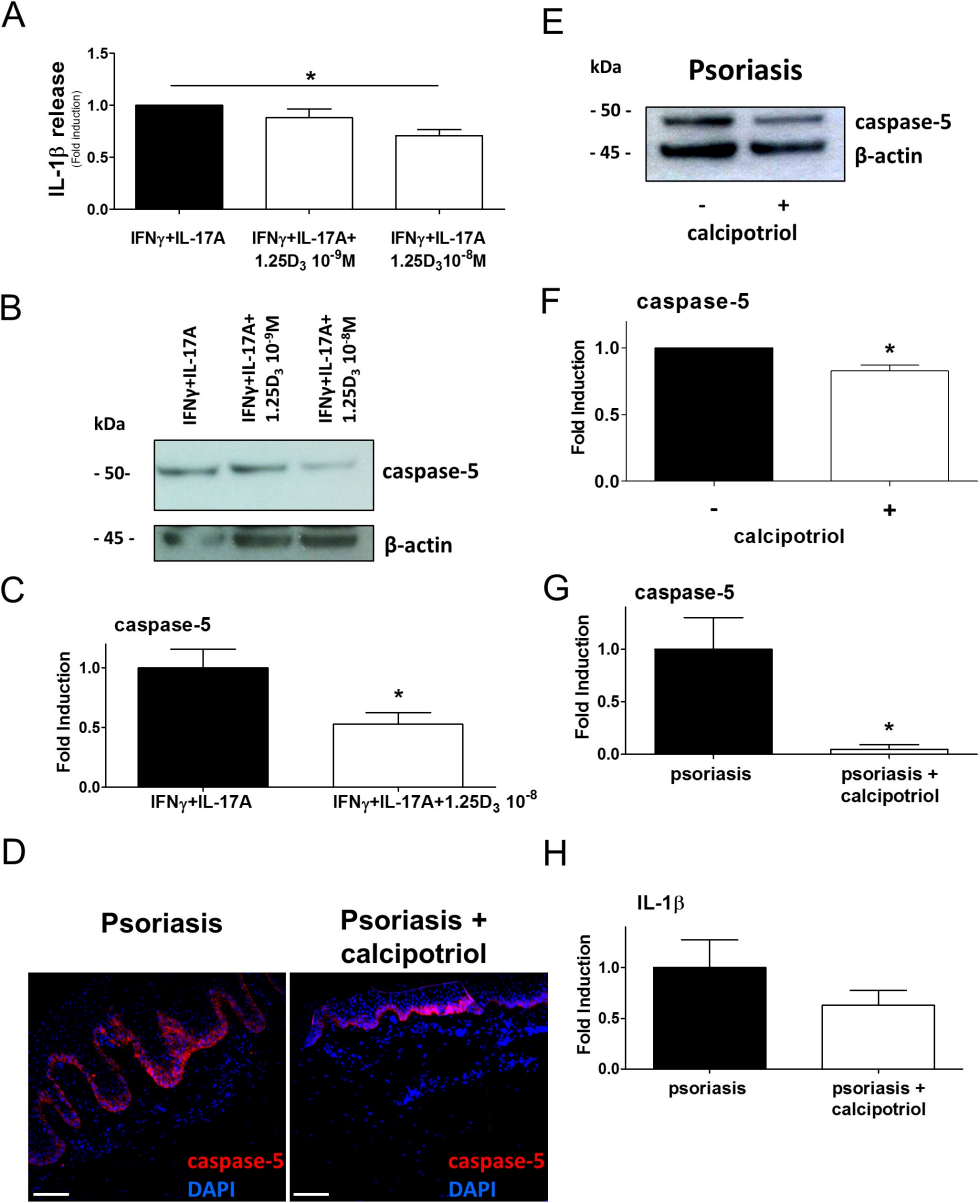
Vitamin D interferes with IL-1β release by epidermal keratinocytes and suppresses caspase-5 expression in epidermal keratinocytes in psoriasis. A, dsDNA-transfected keratinocytes stimulated with IFNγ, IL-17A, and the vitamin D-dependent IL-1β release was analyzed by ELISA, n = 4. B, Keratinocytes stimulated with IFNγ, IL-17A and vitamin D-dependent caspase-5 levels were detected by immunoblotting, n = 3. C, Vitamin D-dependent regulation of caspase-5 in keratinocytes stimulated with IFNγ, IL-17A and analyzed by RTqPCR and normalized to β-actin. Data represent mean + SEM *, p < 0.05 determined by Student’s *t* test, n = 9. D, Representative immunofluorescent staining of caspase-5 in psoriatic skin was reduced after calcipotriol treatment, scale bar = 50 μm. Skin sections of three psoriatic patients were examined. E, F, Representative caspase-5 immunoblotting of caspase-5 levels in skin lysates from psoriatic patients reduced after calcipotriol treatment, protein levels were quantified by densitometry versus β-actin. Data represent mean + SEM, *, *p* < 0.05 determined by Student’s *t* test. Skin lysates of three psoriatic patients were examined. G, H, Regulation of caspase-5 and IL-1β levels in skin lysates from psoriatic patients after calcipotriol treatment as analyzed by RTqPCR and normalized to PBGD. Data represent mean + SEM *, *p* < 0.05; **, *p* < 0.01 determined by Student’s *t* test, n = 3.

Based on data above, topical interference with caspase-5 mediated IL-1β production in lesional psoriatic skin, such as by anti-inflammatory anti-psoriatic vitamin D analogues, might be of therapeutic interest. To test this in a mixed psoriatic micro-milieu, IFNγ and IL-17A primed keratinocytes were transfected with dsDNA, and the IL-1β release was measured dependent on hormonally active vitamin D (calcitriol, 1,25 D_3_) (Fig 5A). In this environment, co-treatment with 1,25 D_3_ reduced the IL-1β release by keratinocytes dose-dependently (up to 0.7-fold). Data further showed that increasing concentrations of 1,25 D_3_ interfered with the cytokine-induced production of caspase-5 (Fig 5B), which is regulated on transcriptional level (0.5-fold) (Fig 5C). However, the expression of caspase-1, NLRP1, or IL-1β were not affected by 1,25 D_3_ in keratinocytes (data not shown).

Vitamin D analogues are standard therapeutics for chronic inflammatory diseases, such as psoriasis [31]. Based on our data, we hypothesized that calcipotriol suppresses caspase-5 expression in the epidermis of psoriatic plaques. Corresponding skin sections of psoriasis showed a reduced epidermal thickening in response to calcipotriol which is accompanied by an attenuated caspase-5 staining in the epidermis (Fig 5D). This was further supported by immunoblot analysis from corresponding skin biopsies showing reduced caspase-5 levels in psoriasis by calcipotriol (0.8-fold) (Fig 5E and 5F). The calcipotriol-mediated suppression of caspase-5 mRNA levels in the psoriatic tissue suggests a transcriptional regulation, which was also detected in 1,25 D_3_ -treated keratinocytes (Fig 5C and 5G). Similar to caspase-5, associated NLRP1 was also suppressed, IL-1β-levels reduced (0.6-fold, p = 0.16), whereas caspase-1 levels remained unaffected (Fig 5H, data not shown). Together, data suggest that targeting caspase-5 may contribute to the anti-inflammatory effects of vitamin D that could be relevant for treatment of Th17-mediated autoinflammatory diseases with caspase-5 overexpression, such as psoriasis or lupus erythematosus (S4 Fig, S5 Fig), or others with proposed NLRP1 inflammasome activity[20, 21].

## Discussion

Here, we observed elevated epidermal expression and increased caspase-5 activation in psoriasis. In epidermal keratinocytes, the intrinsic danger signals cytosolic DNA and the antimicrobial peptide psoriasin (S100A7) were identified as disease-relevant triggers for caspase-5 activity and IL-1β production in a Th1/Th17 environment. Patients suffering from psoriasis have lower vitamin D levels [32], and we observed that application of vitamin D suppresses caspase-5 in keratinocytes in psoriasis. Current studies link IL-1β production and Th17-mediated effects in autoimmune diseases, including psoriasis, and these observations could contribute to the developing understanding of their pathogenesis and therapeutic interference [2, 33, 34].

Inflammatory caspase-5 and associated IL-1β production have been found predominantly in highly immunogenic tissues with close contact to pathogens, such as in leukocytes and in some epithelial cell types [35–38]. There are controversial studies about the expression of caspase-5 in resident skin keratinocytes [12, 39]. Here, we demonstrated that caspase-5 is constitutively expressed in the epidermis of normal skin and functionally active in cultured epidermal keratinocytes.

In the skin, keratinocytes are a major source of IL-1β but the mechanisms of IL-1β production via caspase-5 in a psoriatic Th1/Th17-environment remain unknown [5]. So far, caspase-1 has been identified active and linked to IL-1β maturation in psoriasis [5, 12]. This study showed that caspase-5 is induced in the psoriatic epidermis and activated in psoriatic skin lesions. A scattered single cell stain in the psoriatic dermis might be indicative for caspase-5 expressing immune cells, such as macrophages, which could contribute to the caspase-5 levels detected in lesional psoriasis, also after vitamin D treatment (not shown) [36, 40, 41].

Among the investigated key cytokines relevant in psoriasis, IFNγ was detected as the main inducing factor for inflammatory caspase-5 besides caspase-1 in keratinocytes, whereas IL-17A had an amplifying effect.

Psoriasin (S100A7) was originally identified as antimicrobial peptide in psoriatic keratinocytes, however additional innate functions relevant for epithelial inflammation have recently been uncovered [42, 43]. Our results suggest that psoriasin might further be an intrinsic regulator of IL-1β production in epidermal keratinocytes through regulation of inflammatory caspase-5 and caspase-1 in IFNγ and IL-17A mediated inflammation. Compared to other antimicrobial peptides, such as cathelicidin (LL-37), psoriasin is not able to condense free DNA to influence inflammasome activation (Michel Gilliet, personal communication [44]).

Increasing evidence connects the NLRP1 inflammasome to the pathogenesis of skin-associated autoimmune diseases, such as vitiligo, lupus erythematosus, pemphigus vulgaris, and psoriasis [15, 17, 20, 45]. Compared to other inflammasome complexes, such as NLPR3 and AIM2 expressed in keratinocytes, NLRP1 can activate caspase-5 besides inflammasome ubiquitous caspase-1 [8, 11]. The suppression of caspase-1 recipient NLRP3 and AIM2 by the investigated Th1/Th17 setting in keratinocytes suggests that the steady IL-1β activity is co-sustained by unaffected NLRP1 and induced caspase-5 and caspase-1activity. Thus, besides other inflammasomes active in keratinocytes, NLRP1 is able to contribute to the IL-1β production, particularly in the presence of IL-17A.So far, certain microbial components have been identified to activate the NLRP1 inflammasome, such as muramyl dipeptide (MDP), anthrax lethal factor (LF) from *Bacillus anthracis*, and LPS [13, 14, 36]. Recently, free cytosolic DNA has been found in psoriatic keratinocytes, which can activate caspase-1 via the DNA-sensing inflammasome AIM2 [9, 23]. Here, we could show a DNA-dependent activation of IL-1β in keratinocytes, which is mediated by the NLRP1 and associated caspase-5 and caspase-1. In comparison to previous studies, we were able to detect NLRP1-dependent caspase-5 and caspase-1 cleavage in keratinocytes, which is consistent with reports in other cell types [8, 11, 46]. Our observations on an enhanced caspase-5 regulation in the presence of IL-17A and NLRP1 inflammasome activity in keratinocytes suggests a functional contribution for epidermal IL-1β production in psoriasis and likely other diseases, where Th17 may also contribute, such as lupus erythematosus. The pleiotropic functions of IL-1β produced by epidermal keratinocytes exert local inflammatory effects in the skin, such as release of cytokines and Th17- immunocyte chemotaxins [47]. Skin-attracted and activated immune cells in turn amplify the local inflammation by Th17 cytokine release [48] and subsequent epidermal IL-1β induction as proposed here. This suggests that targeting of local IL-1β production in the skin contributes to the reduction of the local inflammatory phenotype, such as directly through regulation of inflammasomes in the epidermis or through interference with the adjacent Th1/Th17 micro-milieu.

Vitamin D therapeutic effects have been continuously evaluated for numerous chronic inflammatory diseases and cancers [49, 50]. Topical vitamin D analogues are a hallmark in the treatment of psoriasis by affecting skin inflammation through various mechanisms, including the local reduction of Th17 immunocytes [51]. Previous studies underscore the importance of IL-1β regulation in keratinocytes and psoriatic skin by calcipotriol [4, 52]. Here, our data indicate that vitamin D further acts as a direct suppressor of caspase-5 and IL-1β production in keratinocytes suggesting a novel anti-inflammatory mechanism relevant in psoriasis and other Th17-driven inflammatory skin diseases.

Together, this study identified disease-intrinsic regulators, triggers and therapeutic mechanisms of caspase-5 dependent epidermal IL-1β production in psoriasis. These observations may provide insides into current therapies and suggest NLRP1and associated caspase-5 as novel targets for Th17-mediated autoimmune diseases.

## Supporting information

S1 Fig dsDNA and IFNγ induced regulation of IL-1β and caspase-5. A,B, Human epidermal keratinocytes were transfected with dsDNA and stimulated with IFNγ, and the expression of IL-1β and caspase-5 were analyzed by RTqPCR and normalized to β-actin. Data represent mean + SEM of three independent experiments performed in triplicates *, p < 0.05; **, *p* < 0.01; ***, *p* < 0.001 determined by ANOVA.
(PDF)

S2 Fig Knock-down efficacy for caspase-1, caspase-5 and psoriasin (S100A7). A-C, Human epidermal keratinocytes were transfected with siRNA targeting caspase-1, caspase-5, psoriasin and non-coding siRNA, and corresponding targets and off-target controls were analyzed by RTqPCR and normalized to β-actin. Data represent mean + SEM, *, p<0.05; **, *p* < 0.01; ***, *p* < 0.001 determined by Student’s *t* test, n = 9.
(PDF)

S3 Fig Th1/Th17 cytokine-dependent inflammasome regulation and NLRP1 knock-down efficacy in epidermal keratinocytes. A, B, NLRP1, caspase-5 and caspase-1 levels in keratinocytes stimulated with IFNγ, IL-17A analyzed by immunoblotting and normalized to β-actin. One of three representative experiments is shown. **C,** Cell lysates of IFNγ and IL17A-treated keratinocytes, transfected with dsDNA and with siRNA targeting NLRP1 (N1) or non-coding siRNA (Co) stained for NLRP1 (exposure time, 5min) and β-actin normalized. Protein levels were quantified by densitometry versus β-actin, n = 3. n refers to the number of repeated experiments with similar results. D, E, Regulation of NLRP3 and AIM2 in IFNγ- and IL-17A -stimulated keratinocytes analyzed by RTqPCR and normalized to β-actin. Data represent mean + SEM of three independent experiments performed in triplicates *, p < 0.05; **, *p* < 0.01; ***, *p* < 0.001 determined by ANOVA.
(PDF)

S4 Fig Schematic summarizing the proposed mechanism of DNA mediated NLRP1 inflammasome activation in keratinocytes in psoriasis and the therapeutic interference by vitamin D. The autoinflammatory Th1/Th17 milieu in psoriasis contains IFNγ and IL-17A, which induces psoriasin (S100A7)-dependent inflammatory caspases-5 over caspase-1 in keratinocytes. Free dsDNA present in the cytosol of psoriatic keratinocytes activates caspase-5 and caspase-1 dependent on NLPR1 and leads to a subsequent IL-1β release. Topical vitamin D/calcipotriol treatment suppresses caspase-5 and NLRP1 regulation in psoriasis and interferes with IL-1β release by epithelial keratinocytes.
(PDF)

S5 Fig Differential regulation of IL-1β, caspase-1 and caspase-5 in chronic inflammatory skin diseases. Expression levels of IL-1β, caspase-1, caspase-5 in healthy skin compared to tissues from patients with atopic dermatitis, lupus erythematosus and lichen planus analyzed by RTqPCR and normalized to PBGD. Data represent mean + SEM, *, *p* < 0.05 determined by Student’s *t* test. Skin lysates of five patients were examined for each inflammatory skin disease.
(PDF)

## Abbreviations

1,25 D3: calcitriol
AIM2: absent in melanoma 2
AMP: antimicrobial peptide
dsDNA: double-stranded deoxyribonucleic acid
IL-1β: Interleukin-1β
LPS: lipopolysaccharide
NLRP: nucleotide-binding oligomerization domain, leucin-rich repeat and pyrin-domains-containing protein
NOD: nucleotide-binding oligomerization domain
NF-Κb: Nuclear factor κB
MDP: muramyl dipeptide
S100: small calcium-binding proteins 100% soluble in ammonium sulfate
siRNA: small-interference ribonucleic acid
