# Supplementary figures and images for: Th17 micro-milieu regulates NLRP1-dependent caspase-5 activity in skin autoinflammation
Source: PLoS One. 2017 Apr 19;12(4):e0175153. doi: 10.1371/journal.pone.0175153 (PMC5396864; doi:10.1371/journal.pone.0175153)

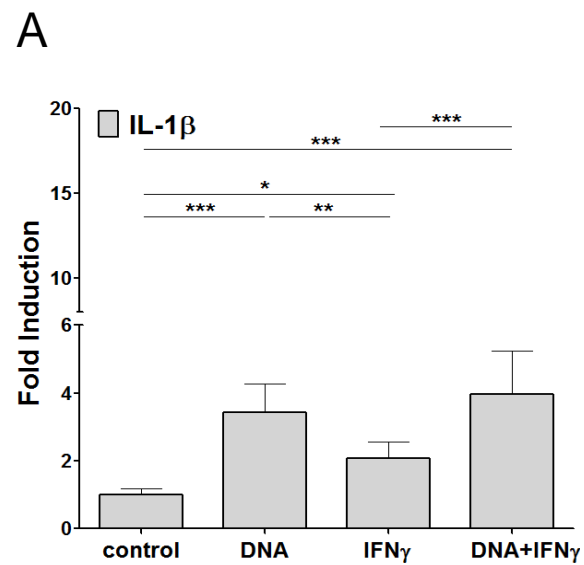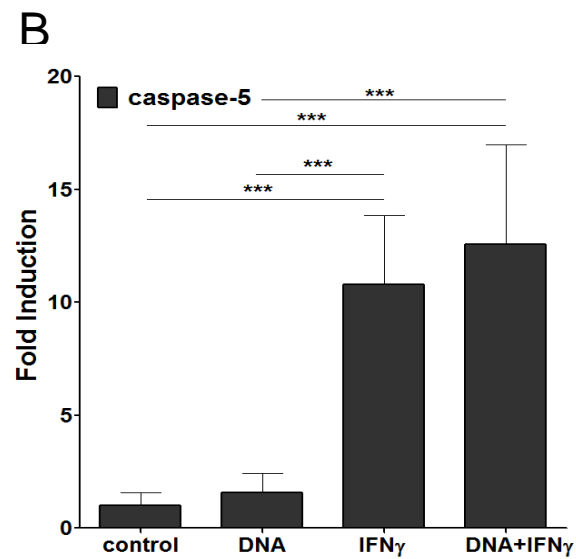

Supplement: S1 Fig — A,B, Human epidermal keratinocytes were transfected with dsDNA and stimulated with IFNγ, and the expression of IL-1β and caspase-5 were analyzed by RTqPCR and normalized to β-actin. Data represent mean + SEM of three independent experiments performed in triplicates *, p < 0.05; **, p < 0.01; ***, p < 0.001 determined by ANOVA. (PDF) [file pone.0175153.s001.pdf]

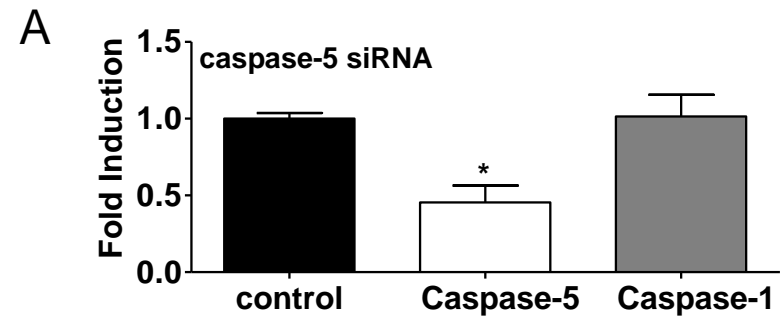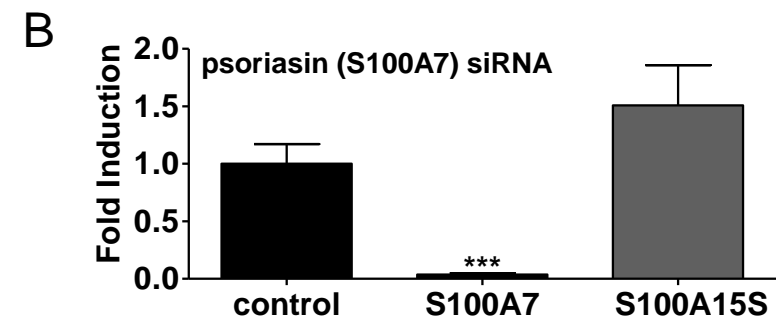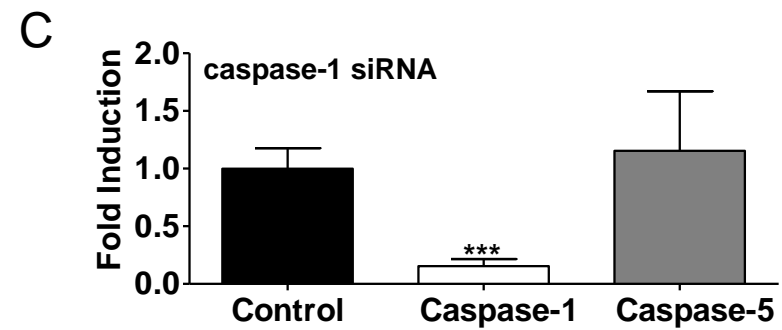

Supplement: S2 Fig — A-C, Human epidermal keratinocytes were transfected with siRNA targeting caspase-1, caspase-5, psoriasin and non-coding siRNA, and corresponding targets and off-target controls were analyzed by RTqPCR and normalized to β-actin. Data represent mean + SEM, *, p<0.05; **, p < 0.01; ***, p < 0.001 determined by Student’s t test, n = 9. (PDF) [file pone.0175153.s002.pdf]

A

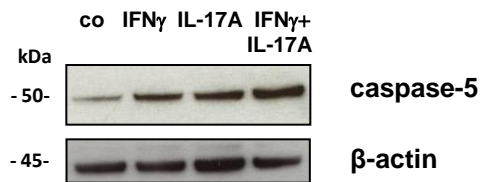

B

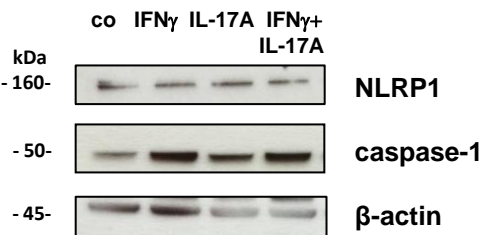

C

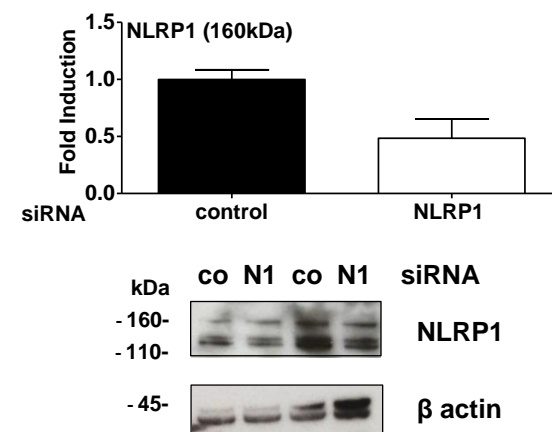

D

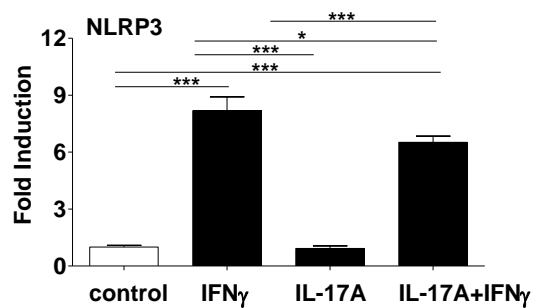

E

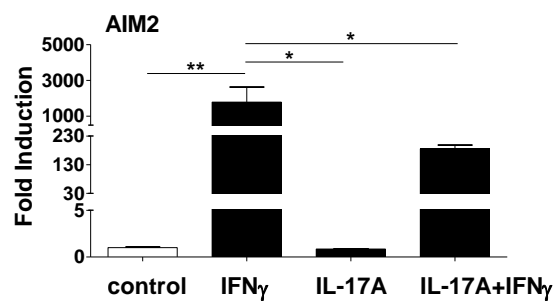

Supplement: S3 Fig — A, B, NLRP1, caspase-5 and caspase-1 levels in keratinocytes stimulated with IFNγ, IL-17A analyzed by immunoblotting and normalized to β-actin. One of three representative experiments is shown. C, Cell lysates of IFNγ and IL17A-treated keratinocytes, transfected with dsDNA and with siRNA targeting NLRP1 (N1) or non-coding siRNA (Co) stained for NLRP1 (exposure time, 5min) and β-actin normalized. Protein levels were quantified by densitometry versus β-actin, n = 3. n refers to the number of repeated experiments with similar results. D, E, Regulation of NLRP3 and AIM2 in IFNγ- and IL-17A -stimulated keratinocytes analyzed by RTqPCR and normalized to β-actin. Data represent mean + SEM of three independent experiments performed in triplicates *, p < 0.05; **, p < 0.01; ***, p < 0.001 determined by ANOVA. (PDF) [file pone.0175153.s003.pdf]

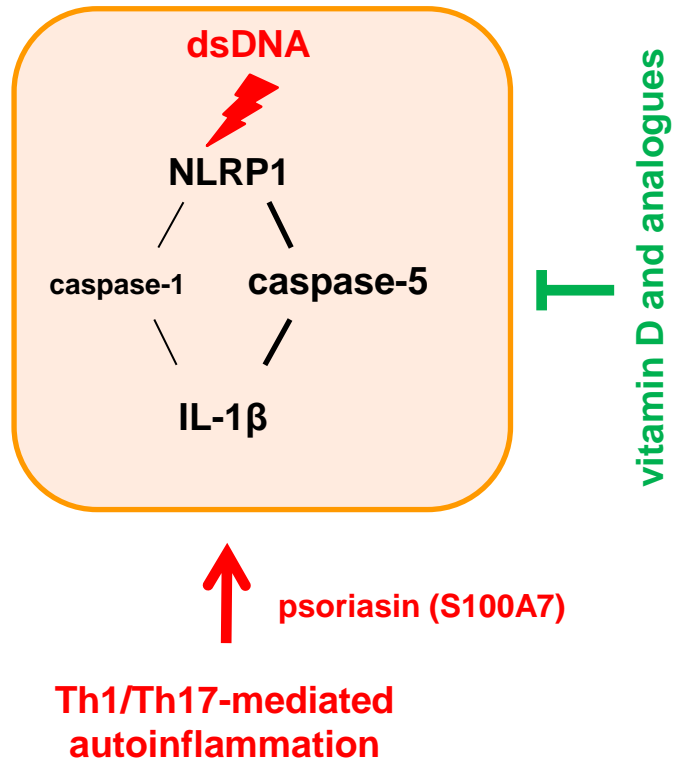

Supplement: S4 Fig — The autoinflammatory Th1/Th17 milieu in psoriasis contains IFNγ and IL-17A, which induces psoriasin (S100A7)-dependent inflammatory caspases-5 over caspase-1 in keratinocytes. Free dsDNA present in the cytosol of psoriatic keratinocytes activates caspase-5 and caspase-1 dependent on NLPR1 and leads to a subsequent IL-1β release. Topical vitamin D/calcipotriol treatment suppresses caspase-5 and NLRP1 regulation in psoriasis and interferes with IL-1β release by epithelial keratinocytes. (PDF) [file pone.0175153.s004.pdf]

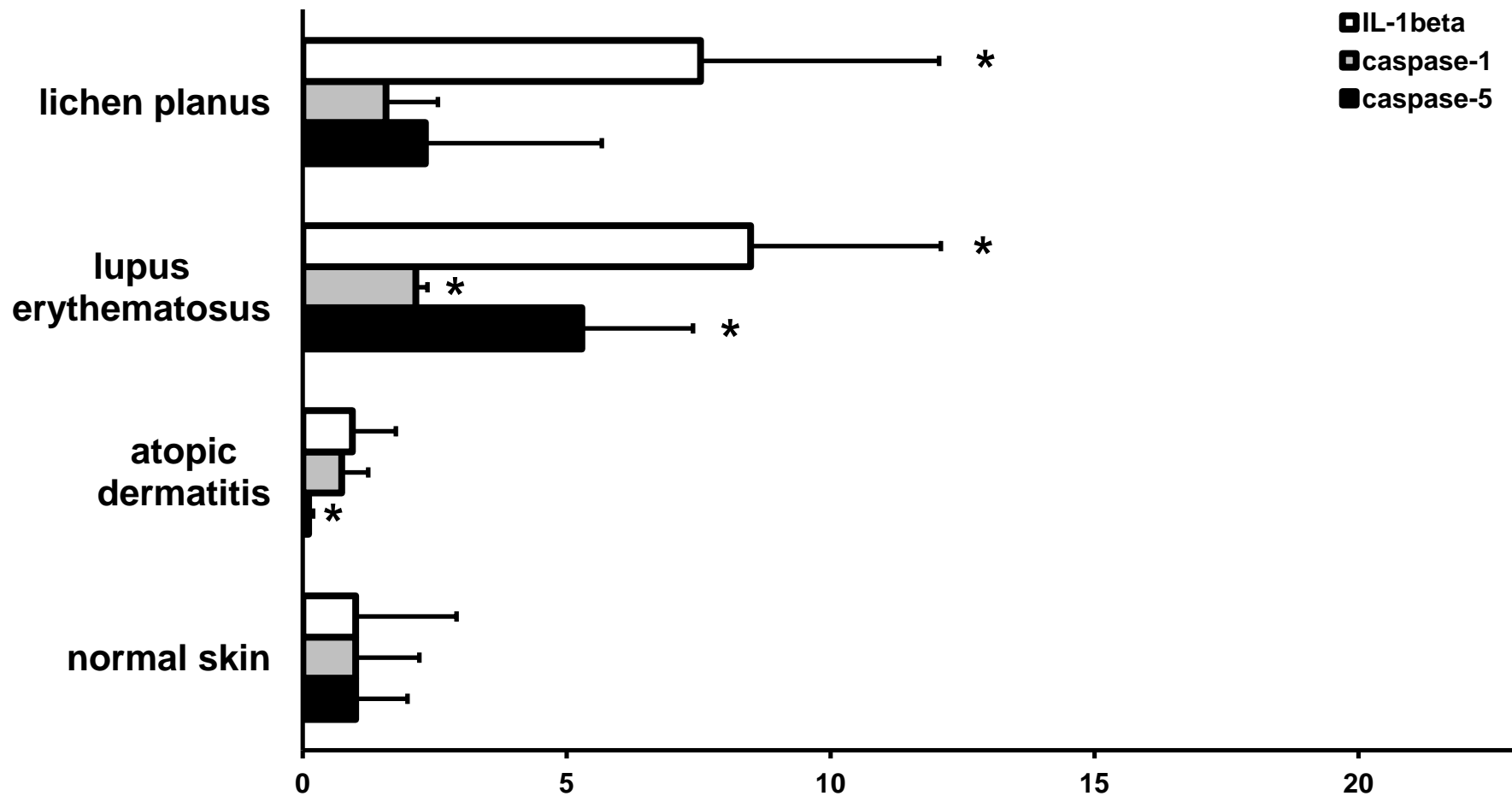

Supplement: S5 Fig — Expression levels of IL-1β, caspase-1, caspase-5 in healthy skin compared to tissues from patients with atopic dermatitis, lupus erythematosus and lichen planus analyzed by RTqPCR and normalized to PBGD. Data represent mean + SEM, *, p < 0.05 determined by Student’s t test. Skin lysates of five patients were examined for each inflammatory skin disease. (PDF) [file pone.0175153.s005.pdf]
